# Pupillometric evidence for the locus coeruleus-noradrenaline system facilitating attentional processing of action-triggered visual stimuli

**DOI:** 10.3389/fpsyg.2015.00827

**Published:** 2015-06-15

**Authors:** Ken Kihara, Tatsuto Takeuchi, Sanae Yoshimoto, Hirohito M. Kondo, Jun I. Kawahara

**Affiliations:** ^1^Graduate School of Science and Engineering, Kagoshima UniversityKagoshima, Japan; ^2^Department of Psychology, Japan Women’s UniversityKanagawa, Japan; ^3^NTT Communication Science Laboratories, NTT CorporationKanagawa, Japan; ^4^United Graduate School of Child Development, Osaka UniversityOsaka, Japan; ^5^Department of Psychology, Hokkaido UniversityHokkaido, Japan

**Keywords:** temporal attention, voluntary action, locus coeruleus-noradrenaline system, pupillometry, rapid serial visual presentation

## Abstract

It has been argued that attentional processing of visual stimuli is facilitated by a voluntary action that triggers the stimulus onset. However, the relationship between action-induced facilitation of attention and the neural substrates has not been well established. The present study investigated whether the locus coeruleus-noradrenaline (LC-NA) system is involved in this facilitation effect. A rapid serial visual presentation paradigm was used to assess the dynamics of transient attention in humans. Participants were instructed to change a digit stream to a letter stream by pressing a button and specifying successive targets of four letters. Pupil dilation was measured as an index of LC-NA function. Accuracy of target identification was better when the temporal delay between participants’ key press and target onset was 800 ms than when targets appeared just after the key press or when targets appeared without key press. Accuracy of target identification was positively correlated with both the peak amplitude of pupil dilation and the pupil size at the time of the key press. These results indicate that target identification in the visual task is closely linked to pupil dilation. We conclude that the LC-NA system plays an important role in the facilitation of transient attention driven by voluntary action.

## Introduction

We interact with the environment around us on a daily basis in order to achieve various goals. Moreover, the visual events that appear in this environment are often triggered by our voluntary actions. The triggering of a visual stimulus voluntarily may modulate our ability to allocate attention toward a particular point in time, or temporal attention, thereby contributing to perceptual enhancement (Hommel et al., 2001; Stock and Stock, 2004). The attentional blink paradigm is useful in the examination of limits of temporal attention for focusing on a series of visual events (Raymond et al., 1992). Typically in an attentional blink paradigm, two targets are embedded in a rapid serial visual presentation (RSVP) stream of non-targets and viewers must identify both targets. When these targets are separated in a stream by less than 500 ms, identification of the first target impairs processing of the second target (i.e., the attentional blink deficit). The attentional blink deficit is generally considered to result from a failure in temporal orienting of attention to the second target after processing the first target (see Martens and Wyble, 2010, for a recent review). In the context of the attentional blink, transient attention which develops rapidly after onset of a visual target, reaches a peak around 100 ms, and decays quickly thereafter (Weichselgartner and Sperling, 1987; Müller and Rabbitt, 1989; Nakayama and Mackeben, 1989). It is considered to be involved in the limitation of the temporal attention (e.g., Olivers and Meeter, 2008). Notably, Kihara and Kawahara (2011) demonstrated that when participants voluntarily triggered the appearance of the first target, the identification accuracy of both first and second target increased. This finding suggests that transient attention driven by the onset of the first target is facilitated by voluntary triggering of the first target, enhancing the first-target processing itself. This permits a rapid orienting of temporal attention from the first to the second target, thus reducing the subsequent attentional blink deficit. However, the neurobiological mechanisms responsible for this attentional facilitation of visual processing triggered by voluntary actions have remained unclear.

Here we postulate that the locus coeruleus-noradrenaline (LC-NA) system is responsible for this attentional facilitation. It is known that the LC-NA system plays an important role in the enhancement of transient attention (for reviews, see Berridge and Waterhouse, 2003; Aston-Jones and Cohen, 2005; Corbetta et al., 2008; Sara and Bouret, 2012). The LC neuronal responses in monkeys are selectively elicited within 100 ms by successful detection of the onset of visual targets; such phasic responses do not occur with missed targets and distractors (Aston-Jones et al., 1994; Clayton et al., 2004). The NA levels in the parietal cortex are induced by LC phasic responses (Foote and Morrison, 1987), and they result in the facilitation of transient attention. In fact, it has been claimed that the LC-NA system is involved in the attentional blink deficit which is modulated by transient attention (Nieuwenhuis et al., 2005).

Previous findings lead to the expectation that attentional processes are facilitated by a voluntary action prior to a visual stimulus, and that the LC-NA system is involved in these processes. However, there is no empirical evidence for a relationship between the voluntary triggering of the visual stimuli and activation of the LC-NA system. To address this, the present study measured pupil diameters in order to identify whether attention processes are affected by the LC-NA system because pupil dilation reflects a phasic response of LC neurons (Koss, 1986; Aston-Jones and Cohen, 2005; Samuels and Szabadi, 2008; Murphy et al., 2011; Laeng et al., 2012; Eldar et al., 2013). The firing rate of LC neurons in monkeys is highly correlated with changes in pupil diameter (Rajkowski et al., 1993). Furthermore, a number of previous studies have demonstrated that the LC-NA system contributes to performance of attentional tasks, as indexed by pupil measurement (for a review, see Laeng et al., 2012). Accordingly, it is reasonable to assume that involvement of the LC-NA system in attentional facilitation, as reflected in pupil dilation, can be observed during a voluntary action that is directed toward a future visual stimulus.

The purpose of the present study was to investigate whether the LC-NA system is involved in the facilitation of transient attention of visual stimuli triggered by voluntary actions, using the pupillary response as a dependent variable. As mentioned earlier, previous studies indicate that transient attention is critical for reporting visual targets presented among an RSVP of to-be-ignored distractors (Olivers and Meeter, 2008; Martens and Wyble, 2010). Weichselgartner and Sperling (1987) used a simple task in which a set of four consecutive targets appeared in an RSVP stream to investigate the nature of transient attention. In this task, via pressing labeled keys, participants reported the identities of four successive targets embedded in a rapid stream of visual non-targets. A typical result of this type of task is that the first target is correctly reported more often than other targets. It is known that up to four items can be maintained within short-term memory (Miller, 1956; Cowan, 2001). If no distractors were presented after the targets, four successive targets could be reported correctly (Olivers et al., 2007). Thus, the differences in the accuracy of reporting targets reflect transient attention, not memory limitations. In addition, previous pupillometric studies have shown the relationship between the activation of the LC-NA system and the identification of a single or multiple target(s) embedded in an RSVP stream (Privitera et al., 2010; Zylberberg et al., 2012). Accordingly, by examining pupillary changes during the RSVP task, we can evaluate the contribution of the LC-NA system to the facilitation of transient attention induced by self-triggered stimulus targets.

In the present study, an RSVP stream consisted of distractors and targets, which respectively, formed two successive strings of items. In the current task, the RSVP stream opened with a variable length string of distractor digits and this was followed by a string of target letters. The experimental conditions required participants to voluntarily trigger, via a key press, the onset of the string of target letters. Finally, we measured changes in participants’ pupillary responses to both the key press and target stimuli. Specifically, we examined the relationship between pupillary change and accuracy of target identification by manipulating the temporal delays between the key press and target onset (Kihara and Kawahara, 2012). We hypothesized that if the LC-NA system contributes to the facilitation of the transient attention induced by self-triggered stimulus targets, then we should find a positive correlation between the accuracy of target identification and maximum pupil diameter size, observed after the voluntary key press. Note that although pupil dilation also occurs if targets need to be memorized for later recall (Peavler, 1974), similar pupil dilation would be observed if the number of to-be-reported targets is the same (Kahneman and Beatty, 1966; Beatty, 1982). A previous study on different patterns of pupillary change suggested that the temporal delays between key press and target onset affect attentional state (Kihara and Kawahara, 2012). Thus, it is possible to separate attention-related pupil dilation from memory-related dilation.

## Materials and Methods

### Participants

Thirty four adults (18 males and 16 females, mean age 22.8 years, range 20–42 years) participated in this experiment. Data from six participants were excluded due to excessive artifacts in their pupil recordings, leaving the data from 28 participants for subsequent analysis. All had self-reported normal or corrected-to-normal vision. Written informed consent was obtained from all participants. This experiment was in accordance with the Declaration of Helsinki and approved by the Committee of Ethics, Chukyo University.

### Apparatus

The experiment was conducted in a darkened room. Stimuli were presented on a 17-inch computer monitor driven at a 60-Hz refresh rate and controlled by MATLAB with the Psychophysics Toolbox (Brainard, 1997; Pelli, 1997). Viewing distance was 57 cm, and head position was maintained by a chin rest. Pupil diameter was recorded using a ViewPoint Eye Tracker (Arrington Research, Inc. Scottsdale, AZ, USA) with a sampling rate of 220 Hz. A video camera and infrared light-emitting diodes were positioned in front of the right eye. The eye tracker was calibrated to each participant at the start of each block of experimental trials. Artifacts and eye blinks were detected by the eye tracking software, and trials in which eye blinks occurred during the time window from the start of the RSVP to the onset of the fourth target were discarded as failing to obtain pupil data.

### Stimuli and Procedures

**FIGURE 1:**
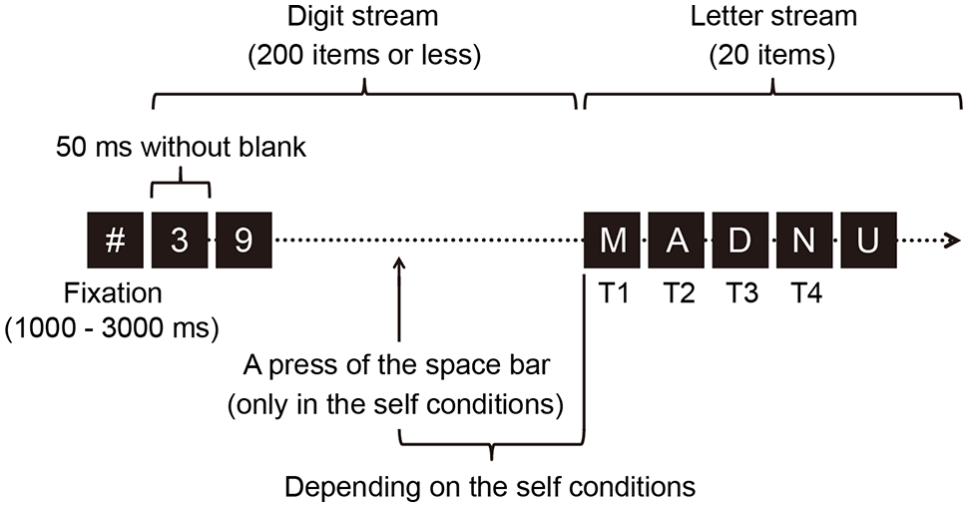
**A schematic illustration of the event sequence in a trial.** Each item in a rapid serial visual presentation (RSVP) stream was presented for 50 ms with no inter-stimulus interval. Each RSVP stream consisted of a digit stream and a letter stream. The procedure involved eight letter-onset conditions. Seven were self conditions in which a participant voluntarily pressed a key to determine the onset time of the switch from the digit sequence to the letter sequence. The voluntary action in these conditions involved a participant voluntarily initiating this switch by pressing the space bar at any arbitrary time (within 10 s). Pre-determined temporal delays occurred between the voluntary key press and the onset of the first capital letter (0–50, 100–150, 200–250, 300–350, 400–450, 600–650, or 800–850 ms). The other condition was an automatic condition, in which the onset time of the letter sequence was automatically determined. In both conditions, participants reported the first, second, third, and fourth targets (T1, T2, T3, and T4, respectively) at the end of the target stream.

**Figure 1** illustrates the sequence of events on a single trial. The RSVP stream consisted of two parts; the first was a stream of 200 (or fewer) digits and the second was a stream of 20 capital letters. The digits were randomly chosen from 0 to 9, with a constraint that the same digit was not presented successively. The capital letters were randomly chosen from A to Z, excluding the letters I, O, and Q. Identical letters were never presented in a trial. Each item subtended a visual angle of approximately 1° × 1°. We used the presentation rate of 50 ms per item because previous studies (Martens et al., 2006; Bowman and Wyble, 2007) have successfully shown minute temporal dynamics in a regular two-target attentional blink procedure at this rate of presentation and our pilot data with this presentation rate demonstrated observable transient attentional effects (Kihara and Kawahara, 2011). The items were colored in dark gray (1.2 cd/m^2^) against a black background (0.3 cd/m^2^).

Letter onset time was also varied as a within-participants letter-onset factor. This variable reflects the temporal delay between the voluntary action and onset time of the letter stream. This variable allows observation of the temporal course of the impact of the voluntary action upon LC-NA system as indexed in changes in a participant’s pupillary response, which was eventually reflected in target reporting scores (Kihara and Kawahara, 2011). According to the preliminary findings, the letter onset variable can be effectively realized with eight letter-onset conditions: seven self conditions and one automatic condition. Each of the eight conditions was presented for one block of forty trials. The order of the block presentation was randomized across participants, except that the automatic condition was not presented as the first block. In the self conditions, pre-determined temporal delays were set between the voluntary key press and the onset of the first capital letter (0–50, 100–150, 200–250, 300–350, 400–450, 600–650, or 800–850 ms); respectively, these correspond to 0, 2, 4, 6, 8, 12, or 16 items between the key press and the first letter. These conditions are labeled 0-, 100-, 200-, 300-, 400-, 600-, or 800- ms condition, respectively. In each trial in each self-condition block, the frame at which participants pressed the key was recorded. In a block of the automatic condition, the first letter appeared automatically at the next frame as that recorded during the immediately preceding self-condition block. This procedure enabled us to minimize the variance in the number of items preceding the first letter between the self- and the automatic-condition blocks for each participant (Kihara and Kawahara, 2012). The order of the trials was randomized.

At the beginning of each block, an instruction relating to key press was displayed on the screen. Each trial began with a hash mark (#) presented for 1000–3000 ms to assist in fixation, followed by a stream of digits. Under the self conditions, participants were asked to voluntarily press the space bar once during the digit stream, within 10 s of the start of the RSVP stream in order to change the stream from digits to letters. When participants pressed the space bar, the RSVP changed with a temporal delay that depended on the conditions. Under the 0-ms delay condition, the first item of the letter stream was presented within 50 ms of the key press, i.e., the first target letter, T1, appeared immediately after the key press that quickly followed a digit item. When participants failed to press the key, the first letter appeared automatically (i.e., the 201st item was the first letter of the letter stream in this case). Under the automatic condition, participants were instructed to refrain from pressing the space bar because the first letter would appear automatically. Once all the items in a stream were presented, participants identified the first four letters by pressing the corresponding keys. Therefore, the first four letters were designed as targets (i.e., T1–T4). A warning message was presented when participants failed to refrain from/perform a response under the automatic/self conditions. Participants were allowed to report the four targets at their own pace.

### Data Analyses

**FIGURE 2:**
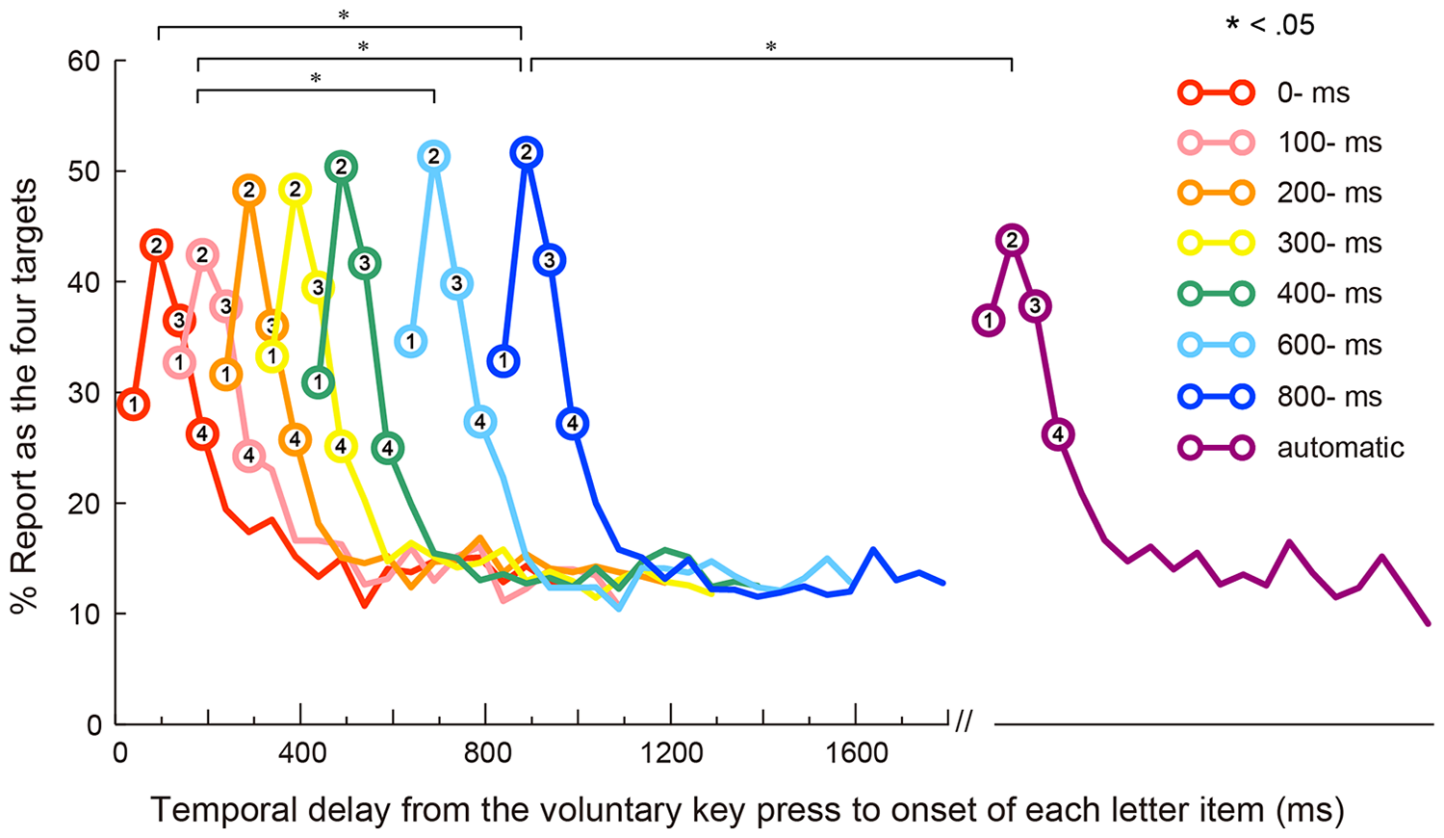
**Behavioral result (significant differences in the report rates of the second target are marked with asterisks: ^∗^*p* < 0.05).** Mean reporting rates of the four targets as a function of temporal delay from the voluntary key press to the onset of each item in each condition. The first four data points (with circles) of each letter-onset condition represent percentages of target reporting. The number on each circle represents each target. The fifth and later data points (without circles) represent percentages of wrong item reporting. (Note: the letter stream was presented automatically without key press in the automatic condition). The differences between the letter-onset conditions are reported for only the second target.

In this experiment, trials in which participants failed to refrain/perform a key press response (0.4% of the total trials) or trials on which the recording of pupil data failed (14.3% of the total trials) were excluded from subsequent analyses. On average, in the self conditions, participants pressed the space bar 1,315 ms (SEM = 110) after the onset of the RSVP stream. We retrospectively counted each letter item reported as one of the four targets regardless of reporting order. Note that the reports of the four targets that can be regarded as correct identifications are indicated by circles in **Figure 2**; therefore, reports of the fifth and later items are regarded as wrong identifications and plotted in this figure as without circles. For example, if a participant reported first, second, fifth, and sixth letters, the first two reports were coded as correct, whereas the latter two were coded as incorrect responses.

Pupillary responses were computed as the percentage increase in pupil area compared with baseline over 100 ms before the onset of the digit stream in each trial (i.e., during presentation of the hash mark). The pre-action period (from the start of an RSVP stream to the voluntary key press) was varied in each trial. In this study, there were not enough trials involving the pre-action period of more than 1000 ms. Thus, to obtain a reliable estimate of ideal pupillary responses relative to the pre-action period, we excluded pupillary data more than 1000 ms before the onset of the key press in the self conditions and the onset of the first letter in the automatic condition, which was based on the immediately preceding self-condition block.

Tukey HSD tests were used as *post hoc* comparisons (alpha-level = 0.05). Pearson correlation coefficients (*r*) were computed to estimate the linear correlation of the behavioral and pupillary data. The Smirnov–Grubbs’ test was used for evaluating outliers.

## Results

The baselines of the pupillary response were not significantly different among the eight letter-onset conditions (i.e., seven self conditions and one automatic condition), confirmed by a one-way repeated-measures analysis of variance (ANOVA), *F*(7,189) = 0.90, n.s., $\eta_{p}^{2}$ = 0.03. **Figure 2** shows the rates of responses reported (as percentages) for items as a function of temporal delay from the voluntary key press to the onset of each item in the eight letter-onset conditions. Because we define targets as the first four letters in the RSVP sequence, the first four data points (with circles) in **Figure 2** indicate correct identification rates of four targets, whereas the fifth and later data points (without circles) indicate the rates of reports of the fifth (or later) items. A two-way repeated-measures ANOVA was conducted on reporting rates with the temporal position of the first four letters and the letter-onset as factors. This analysis yielded significant main effects of the temporal position of the first four letters, *F*(3,81) = 63.29, *p* < 0.001, $\eta_{p}^{2}$ = 0.70, and the letter-onset, *F*(7,189) = 6.22, *p* < 0.001, $\eta_{p}^{2}$ = 0.19. *Post hoc* comparisons revealed that the accuracy for the second target was significantly higher than for other targets. These results indicate that the peak of transient attention developed at around 100 ms after the onset of the first target (Weichselgartner and Sperling, 1987). *Post hoc* comparisons also showed that the mean accuracy for the four targets in the 600- and 800-ms conditions were significantly higher than those in the other letter-onset conditions, suggesting that longer temporal delay between the action and the letter-onset facilitated report of the first four letters. Importantly, the interaction between the letter-onset factor and the temporal position of the first four letters factor was also significant, *F*(21,567) = 5.20, *p* < 0.001, $\eta_{p}^{2}$ = 0.16. Follow up analyses of simple main effects did not show significant effects of the first, *F*(7,189) = 1.15, n.s., $\eta_{p}^{2}$ = 0.04, third, *F*(7,189) = 1.76, n.s., $\eta_{p}^{2}$ = 0.06, or fourth targets, *F*(7,189) = 0.73, n.s., $\eta_{p}^{2}$ = 0.03. However, the second target yielded a significant main effect, *F*(7,189) = 3.99, *p* < 0.001, $\eta_{p}^{2}$ = 0.13. *Post hoc* comparisons revealed that the accuracy of second-target identification was higher for the 600- and 800-ms conditions than the 100-ms condition; accuracy was also higher in the 800-ms condition than in either the 0-ms or automatic conditions. Thus, it appears that a facilitatory effect of transient attention occurred at least 800 ms after the voluntary action.

**FIGURE 3:**
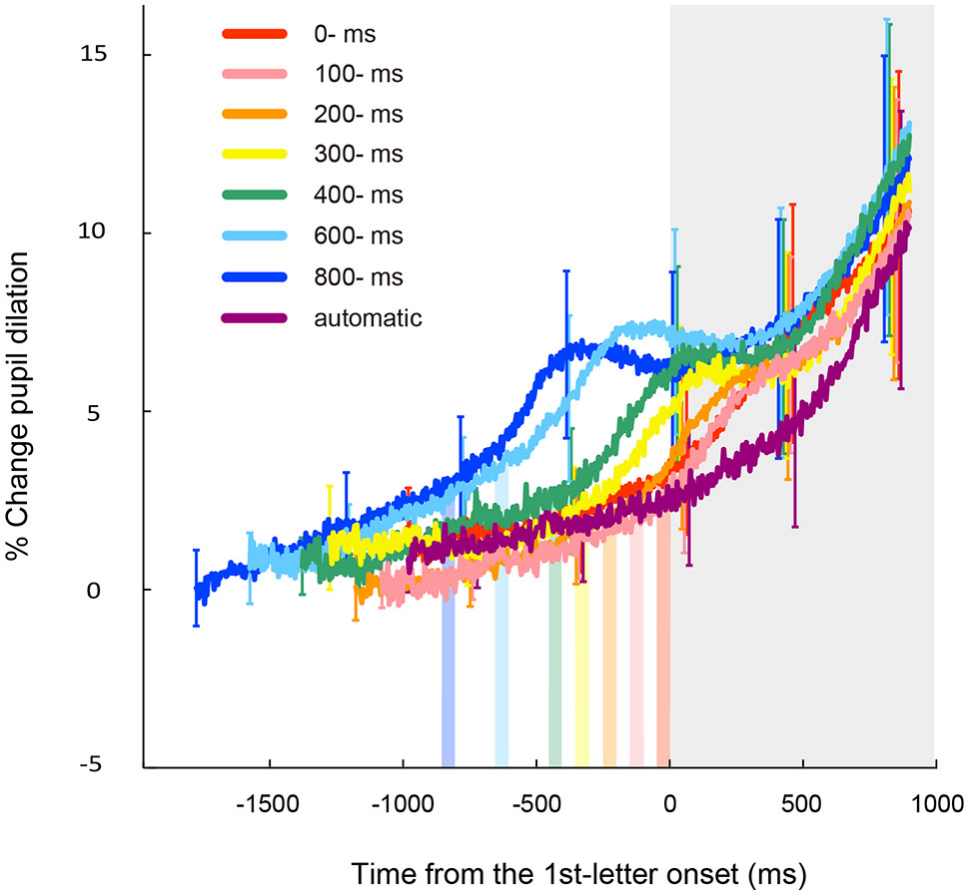
**Results of the time course of mean pupil dilation from the baseline time locked to the onset of the first target for each letter-onset condition.** Each function represents a grand mean of averages of individual participants and is plotted in different colors. Vertical color bars indicate the time window (i.e., the frame duration of 50 ms) in which the voluntary key presses could be executed. Gray area indicates a time window after the onset of the first letter. Error bars indicate 95% confidence intervals.

**FIGURE 4:**
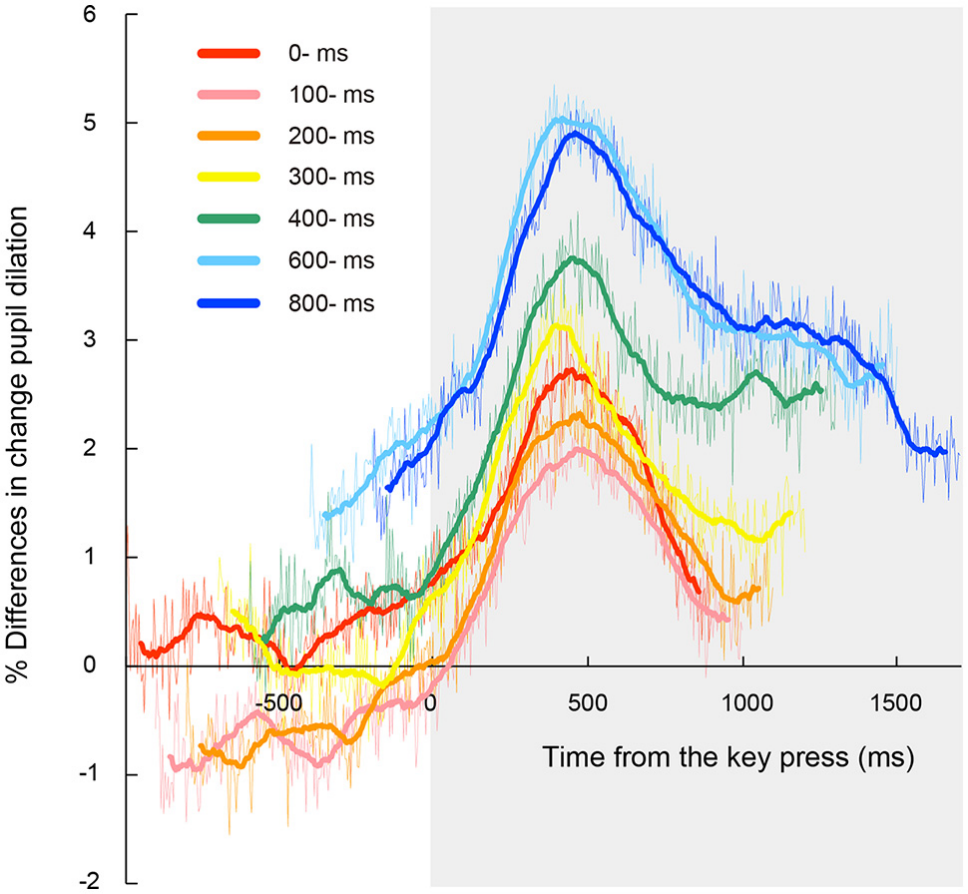
**Time course of mean percentage difference of pupil dilation from the automatic condition time locked to the onset of the voluntary key press for each letter-onset condition.** Thick lines represent data smoothed by averaging a period of 10 samples (i.e., 45 ms) before or after each data point. Thin lines represent the original data before smoothing. Gray area indicates a time window after the onset of the voluntary key press. Note that there was no time course of pupil dilation where the automatic condition could not be subtracted from those in each self condition.

**Figure 3** shows time courses of the observed pupillary dilation, where time is locked to the onset of the first target. Each function represents a grand mean of averages of individual participants. Under all the conditions except the automatic condition, cubic function-like changes were observed: the pupil diameters rapidly increased until about 450 ms after the key press, then leveled off or decreased slightly for a while, and then increased again. It is reasonable to assume that this pattern of pupil changes consists of two components that reflect different cognitive processes. The first component represents a sharper, transient rise associated with the voluntary triggering of the targets, whereas the second component indicates a gradual increase after target onset. The first component is obviously associated with the voluntary key press. On the other hand, the second component was also evident in the automatic condition. Thus, the second component does not depend on voluntary action, but rather on the involvement of the RSVP task in which participants have to memorize and report the visual targets at the end of the RSVP stream. It has been shown that the increase in pupil dilation after the onset of to-be-reported items reflects memory load (Kahneman and Beatty, 1966; Peavler, 1974; Beatty, 1982). Therefore, it is highly likely that the second component of pupil dilation in the present study reflects the memory load associated with the four memorized targets, which were to be reported after the letter stream. We isolated the pupillary responses related to the voluntary triggering by subtracting the pupillary responses in the automatic condition from those in self conditions, and then, sorted the subtracted data based on the onset of the voluntary key press (thin lines in **Figure 4**). To clarify each peak, the time courses of pupil dilation were smoothed by averaging a period of 10 samples (i.e., 45 ms) before and after each data point (thick lines in **Figure 4**). The data of **Figure 4** clearly demonstrate that the voluntary action induced a transient increase in pupil dilation, which peaked at around 450 ms after the action (446, 459, 468, 419, 441, 414, and 455 ms for the 0-, 100-, 200-, 300-, 400-, 600-, and 800-ms delay conditions, respectively).

**FIGURE 5:**
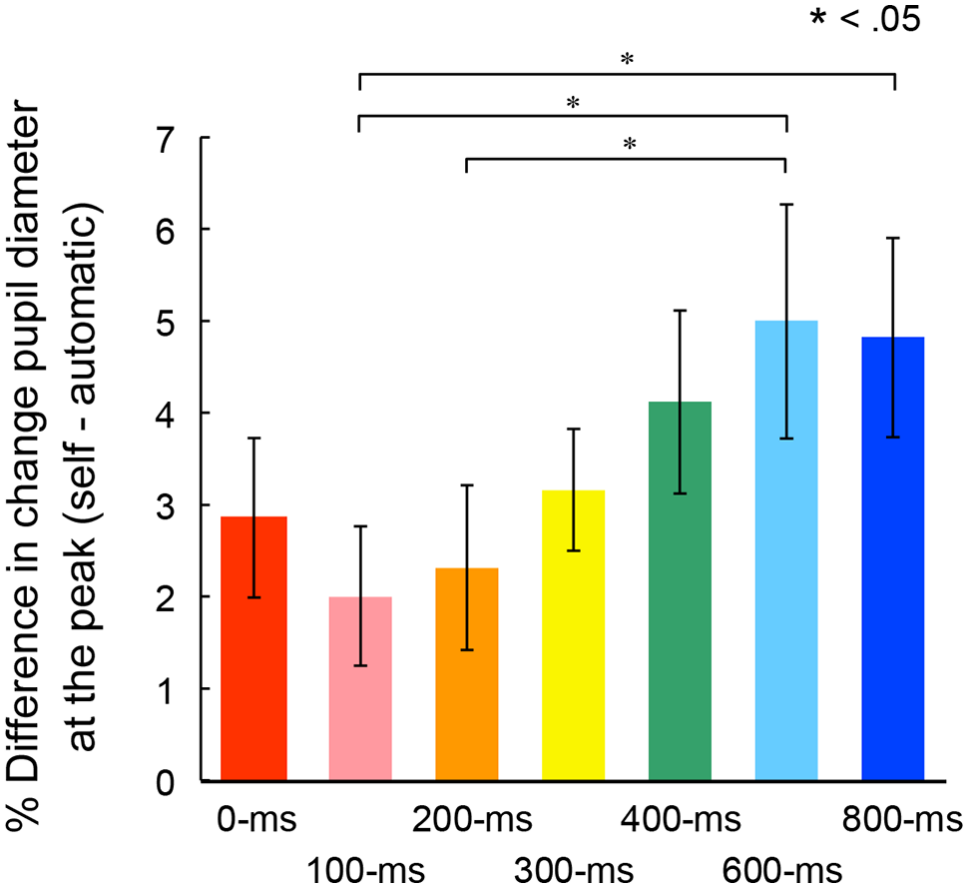
**Mean percentage differences of pupil dilation from the automatic condition at the peak time in each self condition.** Error bars indicate SEM.

**Figure 5** shows mean percentage differences of pupil dilation in the self conditions relative to dilation levels in the automatic condition for the peak time in each self condition. A one-way ANOVA conducted on the mean peak amplitudes revealed a main effect of the self conditions, *F*(6,162) = 3.37, *p* < 0.01, $\eta_{p}^{2}$ = 0.11. *Post hoc* comparisons revealed that peak amplitudes were significantly larger in the 600-ms condition than those in the 100- and 200-ms conditions. The peak amplitude in the 800-ms conditions was also larger than those in the 100-ms condition. These results suggest that the peak in transient pupillary dilation occurred when the targets appeared 600- or 800-ms after the voluntary action.

**FIGURE 6:**
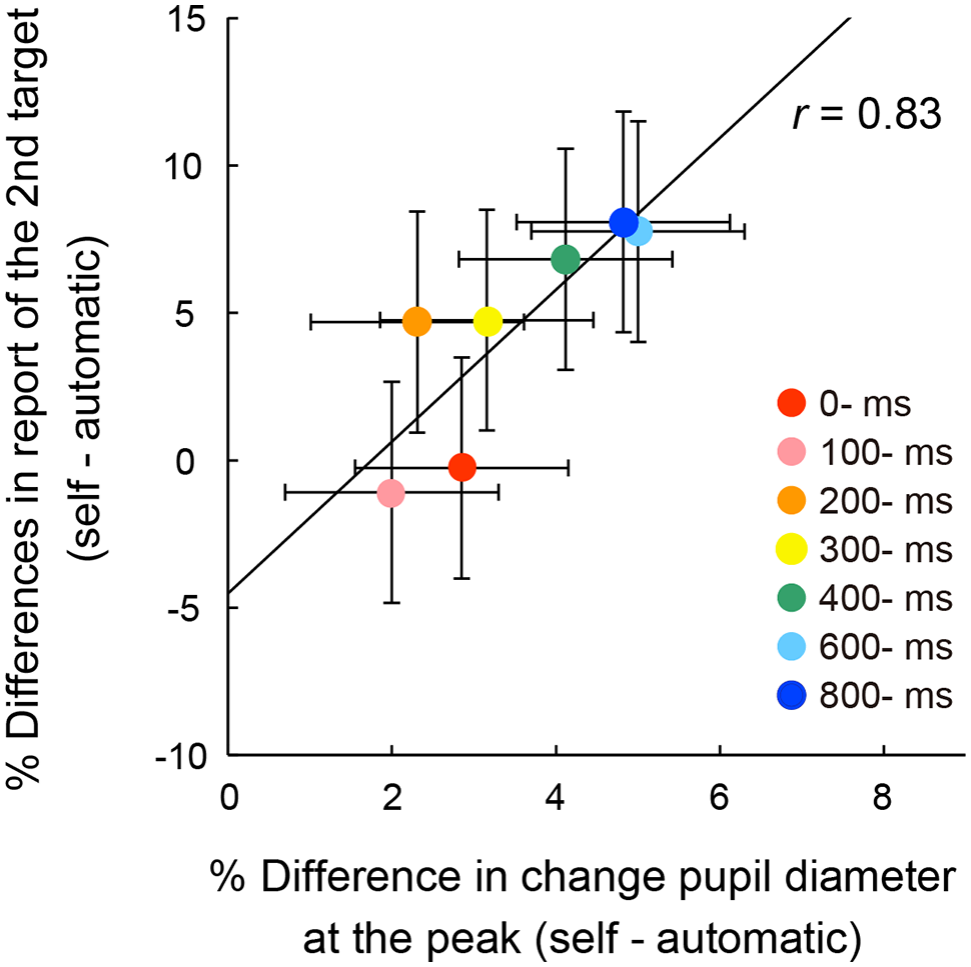
**Correlation between mean percentage differences of reporting rates of the four targets for the second letter item from the automatic condition and differences of peak pupil amplitude from the automatic condition across the self conditions.** The solid line represents the linear regression line. Error bars indicate 95% confidence intervals (Loftus and Masson, 1994).

To examine the relationship between pupil response and action-triggered attentional facilitation, a correlation coefficient was computed between peak pupil dilation and performance of the second target reporting (**Figure 6**). The accuracy for second target responses was calculated by subtracting the report rate for the second targets in the automatic condition from those in each self condition [We used the report rate of the second target rather than the first, as the initial target could reflect inhibition of leading non-targets (Kawahara and Enns, 2009)]. No outliers were identified by the Smirnov–Grubbs test. There was a strong and significant correlation between the peak values and the rates of reporting for the second target (*r* = 0.83, *p* < 0.05, 95% CI [0.21, 0.98]). It is likely that the transient pupillary dilation was associated with the facilitation of transient attention elicited by the voluntary triggering of visual targets. The peak of pupillary dilation and the attentional facilitation increased as the temporal delay increased.

**FIGURE 7:**
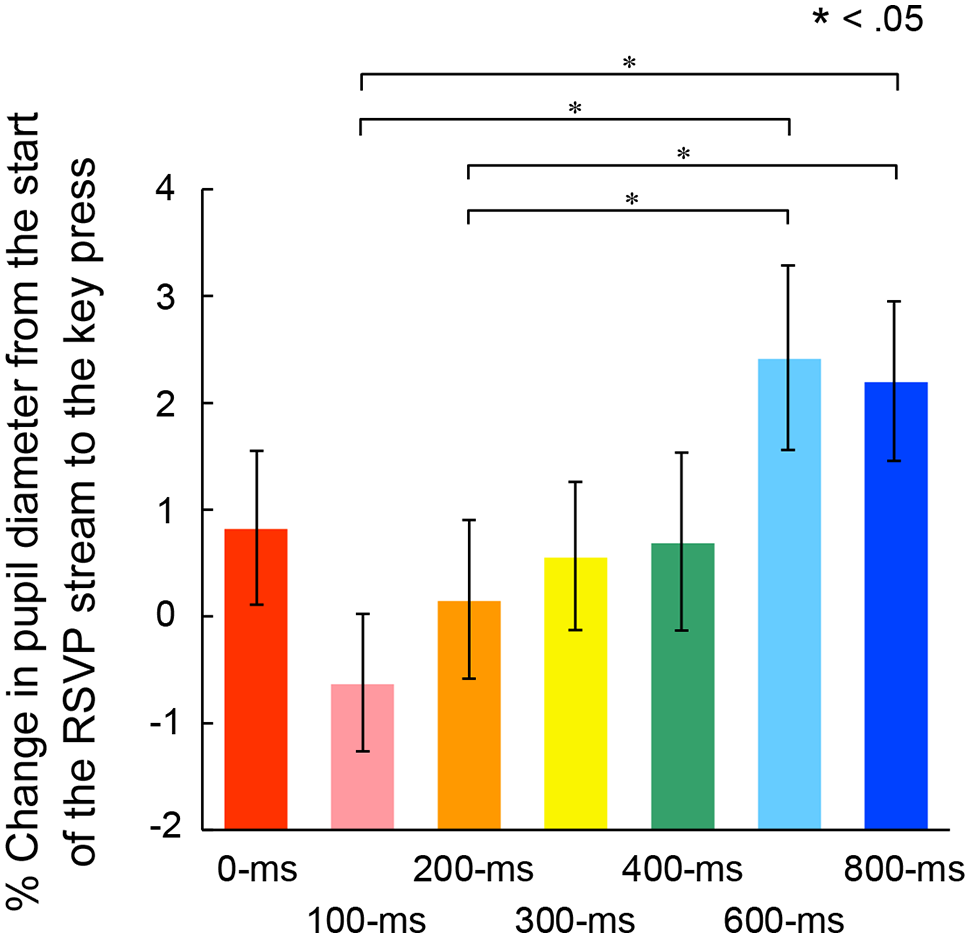
**Mean percentage of pupil dilation from the start of the RSVP stream to the voluntary key press in each self condition.** Error bars indicate SEM.

**FIGURE 8:**
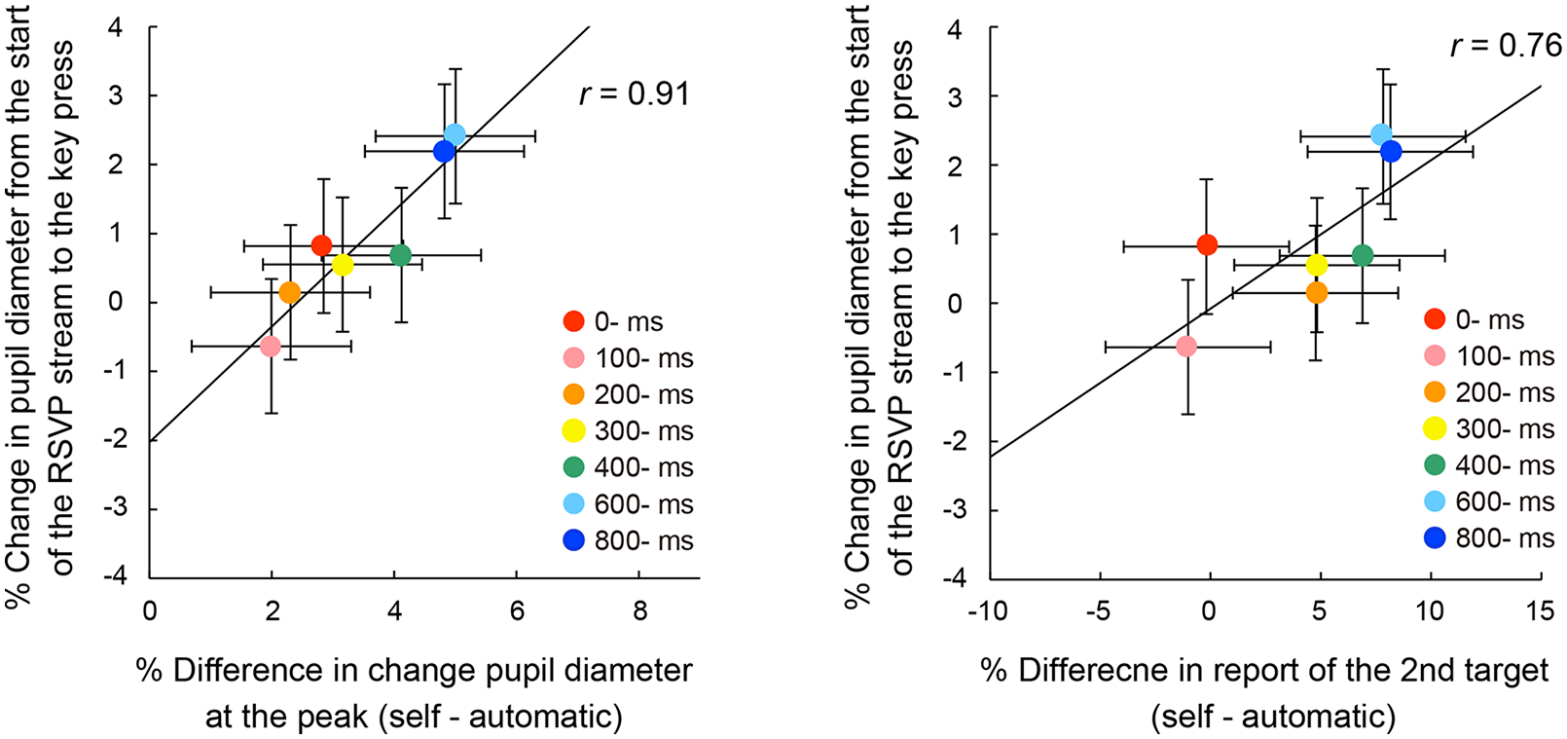
**Correlations between the pupil enlargement ratio from the start of the RSVP stream to the key press and the differences in peak pupil amplitude from the automatic condition **(left)** or the differences of reporting rates for the second letter item from the automatic condition **(right)** across the self conditions.** The solid line represents the linear regression line. Error bars indicate 95% confidence intervals (Loftus and Masson, 1994).

We examined the possibility that the ratio of pupillary dilations began to differ before the voluntary triggering of the targets among the self conditions. In this case, there should be significant differences of the enlargement ratio of pupil diameter at the voluntary key press. **Figure 7** shows the mean enlargement ratio up to the action. A one-way ANOVA indicated a significant main effect of self conditions, *F*(6,162) = 5.21, *p* < 0.001, $\eta_{p}^{2}$ = 0.16. *Post hoc* comparisons revealed that the ratio was significantly larger in the 600- and 800-ms conditions than those in the 100- and 200-ms conditions. In addition, there were significant correlations between the pupil enlargement ratio and the peak pupil amplitude, *r* = 0.91, *p* < 0.05, 95% CI [0.50, 0.99] (**Figure 8**, left panel) and between the pupil enlargement ratio before the key press and the differences of the rates of second target reporting, *r* = 0.76, *p* < 0.05, 95% CI [0.00, 0.96] (**Figure 8**, right panel). Thus, it is reasonable to interpret the observed differences in peak amplitudes of pupil dilation as due mainly to the enlargement of pupil size prior to the voluntary key press.

In summary, the results of behavioral and pupil data indicate that the transient increase in pupil diameter peaked at about 450 ms after a participant’s voluntary action, and it was closely associated with the facilitatory effect of transient attention to visual targets triggered by the action. This transient pupil dilation depended on pupil size at the time of the voluntary action.

## Discussion

The present results demonstrate that the LC-NA system mediates the action-induced facilitation of transient attention as indexed by pupil diameter. In this study, identification accuracy of the second target, embedded in an RSVP stream, was higher than the first, third, and fourth targets, thus indicating the occurrence of transient attention (Weichselgartner and Sperling, 1987). Importantly, identification accuracy of the second target improved when target onsets were triggered by a voluntary key press, suggesting the facilitation of transient attention. This facilitation is observed when the target letters were presented about 600–800 ms after the voluntary action. In addition, the reporting rates of targets were significantly correlated with the peak of pupil dilation at around 450 ms after the voluntary key press across the self conditions. Because pupil dilation reflects activation of the LC-NA system (e.g., Laeng et al., 2012), this finding implies that this system is involved in facilitation of the transient attention driven by action-triggered targets. Thus, we suggest that the LC-NA system leads to the facilitation of transient attention for targets triggered by the action.

We found that the pupil dilation began prior to the action. Previous studies have demonstrated similar results of pupil dilation before a motor response (e.g., Einhäuser et al., 2010; Privitera et al., 2010; Smallwood et al., 2011). This component of the transient pupillary response appears to be a precursor of future voluntary movements (Hupé et al., 2009). Our results also demonstrated that the size of the pupil at the action onset was affected by the temporal delay between the key press and the onset of subsequent target. In this task, participants had to maintain attention to the RSVP stream from the action to the onset of the targets. Accordingly, attentional load would be higher when the maintenance period was longer. It is likely that participants pressed the key in the 600- or 800-ms SOA conditions after sufficient attention was developed as to maintain attention until the appearance of the targets. Task-related decision processes and motor responses are associated with the activation of the LC-NA system (Aston-Jones and Cohen, 2005). Therefore, we believe that the LC neuronal responses are elicited prior to the voluntary action if participants are adequately prepared for the outcomes of these actions, and the facilitation of the transient attention depends on the activation of the LC-NA system related to the motor decisions.

Under the present testing conditions, both the identification accuracies of the second target and the transient increases in pupil diameter were highest when the temporal delay between the key press to the onset of the targets was about 600–800 ms. It is possible that the hidden peaks of these two variables could be observed if the temporal delay were more than 800 ms, although this possibility is questionable because both 600 and 800 ms conditions yielded very similar results. Of course it would be interesting to investigate what length of temporal delay between voluntary action and onset of visual stimulus is optimal, i.e., namely most effective for the facilitation of transient attention. However, this issue is outside of the scope of the present article, which attempts to clarify the relationship between the activation of the LC-NA system and the facilitation of transient attention induced by the voluntary action.

Our findings could possibly be generalized to daily activities where visual events are triggered by a voluntary action. As noted in the introduction, an attentional blink study has demonstrated that it is possible to facilitate attentional processing of voluntary-triggered stimuli embedded in an RSVP stream (Kihara and Kawahara, 2012). Other behavioral studies also suggest a similar facilitation effect. For example, the flash-lag effect, in which a flash is perceived to lag behind a moving object even if both are presented physically aligned (Nijhawan, 2002), has been shown to be reduced when the onset of the flash was triggered by a key press (López-Moliner and Linares, 2006). Temporal orienting of attention plays an important role in the flash-lag effect (Baldo and Klein, 1995; Murakami, 2001; Shioiri et al., 2010; Ichikawa and Masakura, 2013; see also Whitney, 2002, for a review). Interestingly, it has been suggested that the LC-NA system is involved in transient attentional modulation for the flash-lag effect (Bachmann, 2010), as well as for the attentional blink (Nieuwenhuis et al., 2005). Thus, the contribution of the LC-NA system to the facilitation of transient attention to action-triggered visual stimuli is not necessarily limited to RSVP tasks.

It is notable that target accuracy was highest for the second target item, regardless of the temporal delays between the voluntary action and target onset. This pattern of results is frequently observed when people must report multiple items under very short SOAs (Potter et al., 2002; Kawahara and Enns, 2009). Although Weichselgartner and Sperling’s (1987) study showed that the first critical item was reported most frequently, the apparent inconsistency between the present and previous studies may be due to the difference in RSVP rate. Weichselgartner and Sperling (1987) used a presentation rate of 10–12.5 items per second, whereas we adopted 20 items per second. The more rapid rates used in the current task allowed us, to demonstrate the latency of transient attention triggered by target onset. Weichselgartner and Sperling (1987) also reported that the distribution of reportability was bimodal, revealing the existence of both transient and sustained attention. In contrast, the present results yielded a unimodal distribution reflecting transient attention. We assume that the different distributions can be also explained by the difference in RSVP rate. In Weichselgartner and Sperling’s (1987) results, accuracy was highest for the first critical item and next highest for the second item; given RSVP rates, this implies that the transient attention would continue for about 200 ms after the onset of the first target. In this case, during this time window of transient attention, four targets were presented in our task, which used a double-speed RSVP. Therefore, the first three or four targets could be identified as the to-be-identified four targets relatively easily, thus obscuring the second peak of attentional enhancement observed in Weichselgartner and Sperling (1987).

## Conclusion

We investigated the relationship between the LC-NA system and the facilitation of transient attention to visual stimuli whose onset was triggered by a voluntary action by measuring changes in pupil diameter, which reflects the levels of NA released from LC. We found that the reporting rate of a second letter was closely associated with pupil dilation with a peak at around 450 ms after the voluntary action. The peak of pupil dilation depended on the pupil size at the time of the key press, suggesting that the activation of the LC-NA system related to the motor decision contributes to the facilitation of the transient attention. To our knowledge, this is first study to demonstrate that LC-NA system-mediated pupil dilation is related to the facilitation of transient attention driven by action-induced stimuli.

## Conflict of Interest Statement

The authors declare that the research was conducted in the absence of any commercial or financial relationships that could be construed as a potential conflict of interest.

## References

[B1] Aston-Jones G. Cohen J. D. (2005). An integrative theory of locus coeruleus-norepinephrine function: adaptive gain and optimal performance. *Annu. Rev. Neurosci.* 28 403–450. 10.1146/annurev.neuro.28.061604.135709 16022602

[B2] Aston-Jones G. Rajkowski J. Kubiak P. Alexinsky T. (1994). Locus coeruleus neurons in monkey are selectively activated by attended cues in a vigilance task. *J. Neurosci.* 14 4467–4480. 8027789 10.1523/JNEUROSCI.14-07-04467.1994 PMC6577022

[B3] Bachmann T. (2010). “Priming and retouch in flash-lag and other phenomena of the streaming perceptual input,” in *Space and Time in Perception and Action* eds Nijhawan R. Khurana B. (Cambridge: Cambridge University Press) 536–557.

[B4] Baldo M. V. Klein S. A. (1995). Extrapolation or attention shift? *Nature* 378 565–566. 10.1038/378566a0 8524389

[B5] Beatty J. (1982). Task-evoked pupillary responses, processing load, and the structure of processing resources. *Psychol. Bull.* 91 276–292. 10.1037/0033-2909.91.2.276 7071262

[B6] Berridge C. W. Waterhouse B. D. (2003). The locus coeruleus-noradrenergic system: modulation of behavioral state and state-dependent cognitive processes. *Brain Res. Rev.* 42 33–84. 10.1016/S0165-0173(03)00143-7 12668290

[B7] Bowman H. Wyble B. (2007). The simultaneous type, serial token model of temporal attention and working memory. *Psychol. Rev.* 114 38–70. 10.1037/0033-295X.114.1.38 17227181

[B8] Brainard D. H. (1997). The psychophysics toolbox. *Spat. Vis.* 10 433–436. 10.1163/156856897x00357 9176952

[B9] Clayton E. C. Rajkowski J. Cohen J. D. Aston-Jones G. (2004). Phasic activation of monkey locus ceruleus neurons by simple decisions in a forced-choice task. *J. Neurosci.* 24 9914–9920. 10.1523/JNEUROSCI.2446-04.2004 15525776 PMC6730226

[B10] Corbetta M. Patel G. Shulman G. L. (2008). The reorienting system of the human brain: from environment to theory of mind. *Neuron* 58 306–324. 10.1016/j.neuron.2008.04.017 18466742 PMC2441869

[B11] Cowan N. (2001). The magical number 4 in short-term memory: a reconsideration of mental storage capacity. *Behav. Brain Sci.* 24 87–114. 10.1017/S0140525X01003922 11515286

[B12] Einhäuser W. Koch C. Carter O. L. (2010). Pupil dilation betrays the timing of decisions. *Front. Hum. Neurosci.* 4:18 10.3389/fnhum.2010.00018 PMC2831633 20204145

[B13] Eldar E. Cohen J. D. Niv Y. (2013). The effects of neural gain on attention and learning. *Nat. Neurosci.* 16 1146–1153. 10.1038/nn.3428 23770566 PMC3725201

[B14] Foote S. L. Morrison J. H. (1987). Extrathalamic modulation of cortical function. *Annu. Rev. Neurosci.* 10 67–95. 10.1146/annurev.ne.10.030187.000435 3551766

[B15] Hommel B. Müsseler J. Aschersleben G. Prinz W. (2001). The Theory of Event Coding (TEC): a framework for perception and action planning. *Behav. Brain Sci.* 24 849–878. 10.1017/S0140525X01000103 12239891

[B16] Hupé J. M. Lamirel C. Lorenceau J. (2009). Pupil dynamics during bistable motion perception. *J. Vis.* 9 1–19. 10.1167/9.7.10 19761325

[B17] Ichikawa M. Masakura Y. (2013). Effects of consciousness and consistency in manual control of visual stimulus on reduction of the flash-lag effect for luminance change. *Front. Psychol.* 4:120 10.3389/fpsyg.2013.00120 PMC3596863 23504285

[B18] Kahneman D. Beatty J. (1966). Pupil diameter and load on memory. *Science* 154 1583–1585. 10.1126/science.154.3756.1583 5924930

[B19] Kawahara J. Enns J. T. (2009). Selection difficulty and interitem competition are independent factors in rapid visual stream perception. *J. Exp. Psychol. Hum. Percept. Perform.* 35 146–158. 10.1037/a0013164 19170477

[B20] Kihara K. Kawahara J. (2011). Voluntary production of visual items modulates transient attention twice. *J. Vis.* 11 121 10.1167/11.11.121

[B21] Kihara K. Kawahara J. I. (2012). Voluntary triggering of the first target attenuates the attentional blink. *Atten. Percept. Psychophys.* 74 312–321. 10.3758/s13414-011-0233-4 22038666

[B22] Koss M. C. (1986). Pupillary dilation as an index of central nervous system alpha 2-adrenoceptor activation. *J. Pharmacol. Methods* 15 1–19. 10.1016/0160-5402(86)90002-1 2869190

[B23] Laeng B. Sirois S. Gredeback G. (2012). Pupillometry: a window to the preconscious? *Perspect. Psychol.* *Sci* 7 18–27. 10.1177/1745691611427305 26168419

[B24] Loftus G. R. Masson M. E. J. (1994). Using confidence intervals in within-subject designs. *Psychon. Bull. Rev.* 1 476–490. 10.3758/Bf03210951 24203555

[B25] López-Moliner J. Linares D. (2006). The flash-lag effect is reduced when the flash is perceived as a sensory consequence of our action. *Vision Res.* 46 2122–2129. 10.1016/j.visres.2005.11.016 16405940

[B26] Martens S. Munneke J. Smid H. Johnson A. (2006). Quick minds don’t blink: electrophysiological correlates of individual differences in attentional selection. *J. Cogn. Neurosci.* 18 1423–1438. 10.1162/jocn.2006.18.9.1423 16989545

[B27] Martens S. Wyble B. (2010). The attentional blink: past, present, and future of a blind spot in perceptual awareness. *Neurosci. Biobehav. Rev.* 34 947–957. 10.1016/j.neubiorev.2009.12.005 20025902 PMC2848898

[B28] Miller G. A. (1956). The magical number seven plus or minus two: some limits on our capacity for processing information. *Psychol. Rev.* 63 81–97. 10.1037/h0043158 13310704

[B29] Müller H. J. Rabbitt P. M. (1989). Reflexive and voluntary orienting of visual attention: time course of activation and resistance to interruption. *J. Exp. Psychol. Hum. Percept. Perform.* 15 315–330. 10.1037/0096-1523.15.2.315 2525601

[B30] Murakami I. (2001). The flash-lag effect as a spatiotemporal correlation structure. *J. Vis.* 1 126–136. 10.1167/1.2.6 12678607

[B31] Murphy P. R. Robertson I. H. Balsters J. H. O’connell R G. (2011). Pupillometry and P3 index the locus coeruleus-noradrenergic arousal function in humans. *Psychophysiology* 48 1532–1543. 10.1111/j.1469-8986.2011.01226.x 21762458

[B32] Nakayama K. Mackeben M. (1989). Sustained and transient components of focal visual attention. *Vision Res.* 29 1631–1647. 10.1016/0042-6989(89)90144-2 2635486

[B33] Nieuwenhuis S. Gilzenrat M. S. Holmes B. D. Cohen J. D. (2005). The role of the locus coeruleus in mediating the attentional blink: a neurocomputational theory. *J. Exp. Psychol. Gen.* 134 291–307. 10.1037/0096-3445.134.3.291 16131265

[B34] Nijhawan R. (2002). Neural delays, visual motion and the flash-lag effect. *Trends Cogn. Sci.* 6 387–393. 10.1016/S1364-6613(02)01963-0 12200181

[B35] Olivers C. N. L. Meeter M. (2008). A boost and bounce theory of temporal attention. *Psychol. Rev.* 115 836–863. 10.1037/a0013395 18954206

[B36] Olivers C. N. L. Van Der Stigchel S. Hulleman J. (2007). Spreading the sparing: against a limited-capacity account of the attentional blink. *Psychol. Res.* 71 126–139. 10.1007/s00426-005-0029-z 16341546

[B37] Peavler W. S. (1974). Pupil size, information overload, and performance differences. *Psychophysiology* 11 559–566. 10.1111/j.1469-8986.1974.tb01114.x 4415394

[B38] Pelli D. G. (1997). The VideoToolbox software for visual psychophysics: transforming numbers into movies. *Spat. Vis.* 10 437–442. 10.1163/156856897X00366 9176953

[B39] Potter M. C. Staub A. O’connor D. H. (2002). The time course of competition for attention: attention is initially labile. *J. Exp. Psychol. Hum. Percept. Perform.* 28 1149–1162. 10.1037//0096-1523.28.5.1149 12421061

[B40] Privitera C. M. Renninger L. W. Carney T. Klein S. Aguilar M. (2010). Pupil dilation during visual target detection. *J. Vis.* 10 1–14. 10.1167/10.10.3 20884468

[B41] Rajkowski J. Kubiak P. Aston-Jones G. (1993). Correlations between locus coeruleus (LC) neural activity, pupil diameter and behavior in monkey support a role of LC in attention. *Soc. Neurosci. Abstr.* 19 974.

[B42] Raymond J. E. Shapiro K. L. Arnell K. M. (1992). Temporary suppression of visual processing in an RSVP task: an attentional blink? *J. Exp. Psychol. Hum. Percept. Perform.* 18 849–860. 10.1037//0096-1523.18.3.849 1500880

[B43] Samuels E. R. Szabadi E. (2008). Functional neuroanatomy of the noradrenergic locus coeruleus: its roles in the regulation of arousal and autonomic function Part II: physiological and pharmacological manipulations and pathological alterations of locus coeruleus activity in humans. *Curr. Neuropharmacol.* 6 254–285. 10.2174/157015908785777193 19506724 PMC2687931

[B44] Sara S. J. Bouret S. (2012). Orienting and reorienting: the locus coeruleus mediates cognition through arousal. *Neuron* 76 130–141. 10.1016/j.neuron.2012.09.011 23040811

[B45] Shioiri S. Yamamoto K. Oshida H. Matsubara K. Yaguchi H. (2010). Measuring attention using flash-lag effect. *J. Vis.* 10 1–13. 10.1167/10.10.10 20884475

[B46] Smallwood J. Brown K. S. Tipper C. Giesbrecht B. Franklin M. S. Mrazek M. D. (2011). Pupillometric evidence for the decoupling of attention from perceptual input during oﬄine thought. *PLoS ONE* 6:e18298 10.1371/journal.pone.0018298 PMC3064669 21464969

[B47] Stock A. Stock C. (2004). A short history of ideo-motor action. *Psychol. Res.* 68 176–188. 10.1007/s00426-003-0154-5 14685855

[B48] Weichselgartner E. Sperling G. (1987). Dynamics of automatic and controlled visual attention. *Science* 238 778–780. 10.1126/science.3672124 3672124

[B49] Whitney D. (2002). The influence of visual motion on perceived position. *Trends Cogn. Sci.* 6 211–216. 10.1016/S1364-6613(02)01887-9 11983584 PMC3849397

[B50] Zylberberg A. Oliva M. Sigman M. (2012). Pupil dilation: a fingerprint of temporal selection during the “attentional blink”. *Front. Psychol.* 3:316 10.3389/fpsyg.2012.00316 PMC3428810 22973248

